# Detailed Comparison of Acoustic Signals from Rehabilitated and Wild Franciscanas (*Pontoporia blainvillei*) Dolphins

**DOI:** 10.3390/ani14162436

**Published:** 2024-08-22

**Authors:** Gisela Vanina Giardino, Mel Cosentino, Agustina Camila Macchi, Juan Pablo Loureiro, Sergio Rodriguez Heredia, Karina Ceilia Alvarez, Sergio Gabriel Moron, Diego Horacio Rodriguez

**Affiliations:** 1 Instituto de Investigaciones Marinas y Costeras (IIMyC), FCEyN, Universidad Nacional de Mar del Plata-CONICET, Mar del Plata 7600, Argentina; agusmacchi.1995@gmail.com (A.C.M.); dhrodri@gmail.com (D.H.R.); 2 Section for Marine Mammal Research, Department of Ecoscience, Aarhus University, 4000 Roskilde, Denmark; melcos@ecos.au.dk; 3 Fundación Mundo Marino, San Clemente del Tuyú 7105, Argentina; juanploureiro@gmail.com (J.P.L.); papppoblues@gmail.com (S.R.H.); conservacion@fundmundomarino.org.ar (K.C.A.); moron_sergio@hotmail.com (S.G.M.)

**Keywords:** NBHF species, animal communication, bycatch, *Pontoporia blainvillei*, rehabilitation, animal welfare, acoustic monitoring

## Abstract

**Simple Summary:**

Our research focuses on the franciscana dolphin, the most threatened dolphin in the southwestern Atlantic, mainly due to its frequent entanglement in artisanal gillnets. This has led to their classification as vulnerable. To better understand their sounds and communication, we recorded and analysed the sounds of both wild dolphins and those in a rehabilitation centre. We found that neonate dolphins emit sounds that differ from those of juveniles. Interestingly, the sounds made by juvenile dolphins are similar to those of wild dolphins. We also discovered patterns in the way dolphins produce clicks, sounds which are important for feeding and communication. Our findings highlight the need to protect calf dolphins and suggest that it might be possible to create tools to identify dolphins by their age, based on their sounds. This research is important for conservation efforts and could help in creating better protection strategies for these dolphins.

**Abstract:**

The franciscana dolphin is a small, vulnerable species often caught in artisanal gillnets. This study aims to provide a comprehensive assessment of their acoustic capabilities by using advanced equipment to collect a large dataset of wideband, continuous recordings. We examined the detailed acoustic signals of franciscana dolphins, comparing the sounds from rehabilitated dolphins in captivity with those of wild dolphins near fishing nets. Significant differences in acoustic characteristics were found between neonates and older dolphins, with juvenile and wild dolphins showing similar features. For the first time, repetition patterns in click production were identified, highlighting the importance of understanding the context of these sounds in regards to feeding and communication. This study emphasizes the need for detecting neonates for species protection and suggests the potential for developing acoustic classifiers specific to different age groups. Our findings offer valuable insights for conservation efforts and the development of protection strategies for franciscana dolphins.

## 1. Introduction

The franciscana dolphin (*Pontoporia blainvillei*) is a small odontocete found in the coastal waters of Brazil, Uruguay, and Argentina [1]. Their range extends from Espírito Santo in Brazil (18°25′ S-39°42′ W) to the Golfo San Matías in Argentina (43°18′ S-65°06′ W) [2,3]. Even though they are basically coastal, found between the surf zone and 50 m deep waters [4,5], franciscanas are difficult to observe in the wild due to their small size, cryptic colouration, lack of aerial behaviours, and their formation of groups of fewer than six animals [6,7], which also explains why so little is known about them.

The main threat to their survival is being caught in gillnets, with an estimate of at least 400 animals caught annually in Argentine waters [8]. Current abundance estimates are lacking, but it is believed that there are fewer than 20,000 franciscanas, resulting in unsustainable levels of bycatch [9,10]. These numbers led the International Union for Conservation of Nature (IUCN) and the Mastozoological Society of Argentina (SAREM) to list franciscanas as vulnerable [3,11]. Moreover, in Buenos Aires Province, franciscanas were declared a provincial monument (Law 14992, OPDS) to provide them with legal protection and to develop conservation strategies for these creatures. Despite these measures, there are no systematic efforts to monitor the species in Argentina.

Visual methods to monitor franciscanas are limited because of their cryptic behaviour, as well as the muddy nature of the waters they inhabit; moreover, surveys are expensive and are included only as a snapshot in large-scale surveys. Passive acoustic monitoring (PAM) methods, on the other hand, can be deployed for long periods and are not weather or visibility dependent. PAM can be used to investigate their distribution range, density, and potentially, their behaviour [12,13,14,15].The survival of franciscanas, therefore, depends on having an accurate description of the acoustic properties of the sounds they emit to effectively monitor them using PAM systems.

Franciscanas are one of the sixteen species that produce narrow-band and high-frequency (NBHF) clicks, including all porpoises (seven species), both Kogia, all *Cephalorhynchus* (four species), and two *Lagenorhynchus* [16,17,18]. NBHF clicks are remarkably similar in these species, with most of the energy concentrated between 100 kHz and 150 kHz, and a duration of around 100 µs [19]. Some of these species, such as the Commerson’s dolphin (*Cephalorhynchus commersonii*), also emit other sounds, including lower frequency whistles [20]. These NBHF clicks are used for echolocation, as well as for communication, and it is thought that information is encoded in the variations in click production rates. In such cases, the clicks are emitted at much higher rates, known as burst pulses (e.g., in *Lagenorhynchus obliquidens* [21], *Monodon monoceros* [22], *Lissodelphis borealis* [23], and *Neophocaena asiaeorientalis* [24].

The few studies focused on the characteristics of franciscana sounds, either recorded in animals under human care or in the wild, showed that they emit NBHF clicks with a peak frequency around 130 kHz [25,26,27]. Neonates, however, appear to be able to emit echolocation clicks with peak frequencies below 50 kHz [26,28]. Some studies have reported that franciscanas also emit sounds with the energy concentrated in much lower frequencies. For example, Cremer et al. [29] reported whistles with frequencies between 1.6 and 94.6 kHz, recorded mainly from individuals under stress conditions. Barcellos et al. [30] claim that burst pulses exhibit energy concentrations at lower frequencies and wider bandwidths [30,31].

These studies have provided important information about the acoustic characteristics of franciscanas; however, in some cases, the methodology and equipment used were limiting; for example, the sampling frequencies employed were not able to capture the NBHF clicks (e.g., [28]) or the devices used did not record the raw sounds, but only logged data regarding detections (e.g., [32].

In this study, we used equipment and methodologies capable of acquiring a large dataset of wideband, continuous recordings that are essential for a comprehensive assessment of the acoustic capabilities of franciscanas. Therefore, the overall purpose of this study is to provide a detailed assessment of the acoustic parameter of franciscana dolphins. The specific objectives of this study were as follows:

1. To describe and compare the detailed characteristics of the acoustic signals recorded from:
  a. Two neonates (<1 month old) in a rehabilitation centre.
  b. One juvenile (1–2 years old) in a rehabilitation centre.
  c. Wild animals of unknown age classes detected around gillnets in the coasts of Buenos Aires.
2. To identify patterns of repetition rates in click production

## 2. Materials and Methods

**Figure 1:**
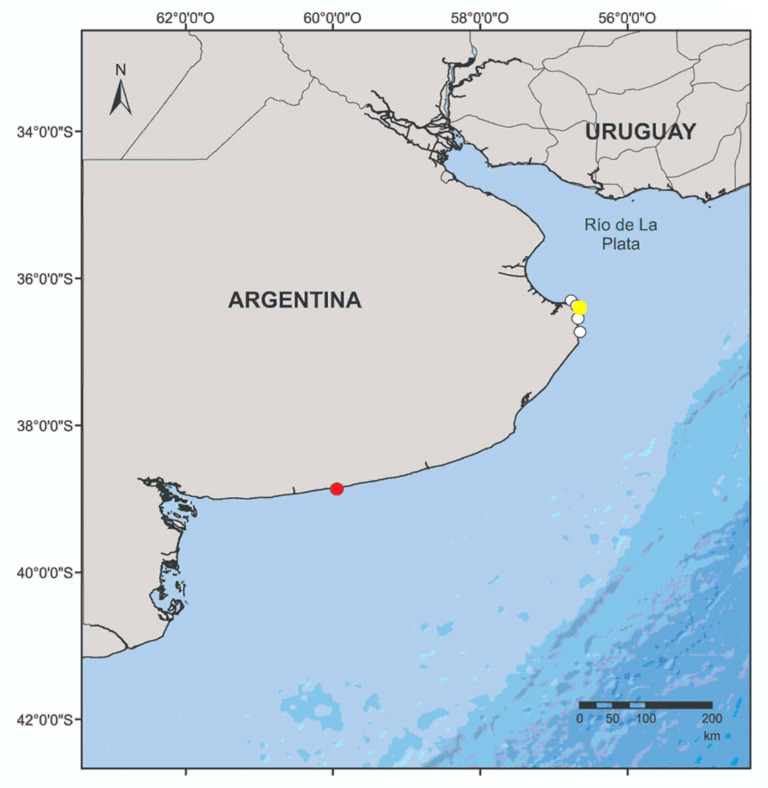
Map of the study area. White circles: location where the neonates and juvenile franciscanas were found after stranding alive. Yellow circle: location of rehabilitation centre. Red circle: location of the gillnets (off Claromecó) where the wild franciscanas were recorded.

The acoustic data used in this study were obtained using wideband recorders from wild animals off of Claromecó (Buenos Aires, Argentina) around static fishing nets and from three live-stranded franciscana dolphins that were taken to the rehabilitation centre of Fundación Mundo Marino (San Clemente del Tuyu), after several unsuccessful release attempts. The strandings occurred in Punta Rasa (juvenile), Las Toninas (neonate 1), and Costa del Este (neonate 2) (Figure 1). The transport of stranded animals to the rehabilitation centre was conducted following the recommendations of the Neonatal Care and Hand-Rearing Protocol–Rescue and Rehabilitation of Franciscana Dolphins [33], the Argentine laws 22.421, 25.052, and 25.052, and the standard ethic criteria established by the Marine Fauna Rescue Network of OPDS (Organismo Provincial Para el Desarrollo Sostenible—Provincial Organism for Sustainable Development, Buenos Aires, Argentina; Resolution 86/10). During the rehabilitation process, veterinary medical procedures were performed, such as ultrasound analysis, blood tests, and faecal matter analysis, as described in the work of Kolesnikovas [34].

### 2.1. Data Collection

All recordings were produced using a SoundTrap300 HF (ST, Ocean Instruments, Auckland, New Zealand), at a sampling frequency of 576 kHz. The ST exhibited a sensitivity of −176.4 dB re 1 V/µPa.

#### 2.1.1. Neonate 1

**Figure 2:**
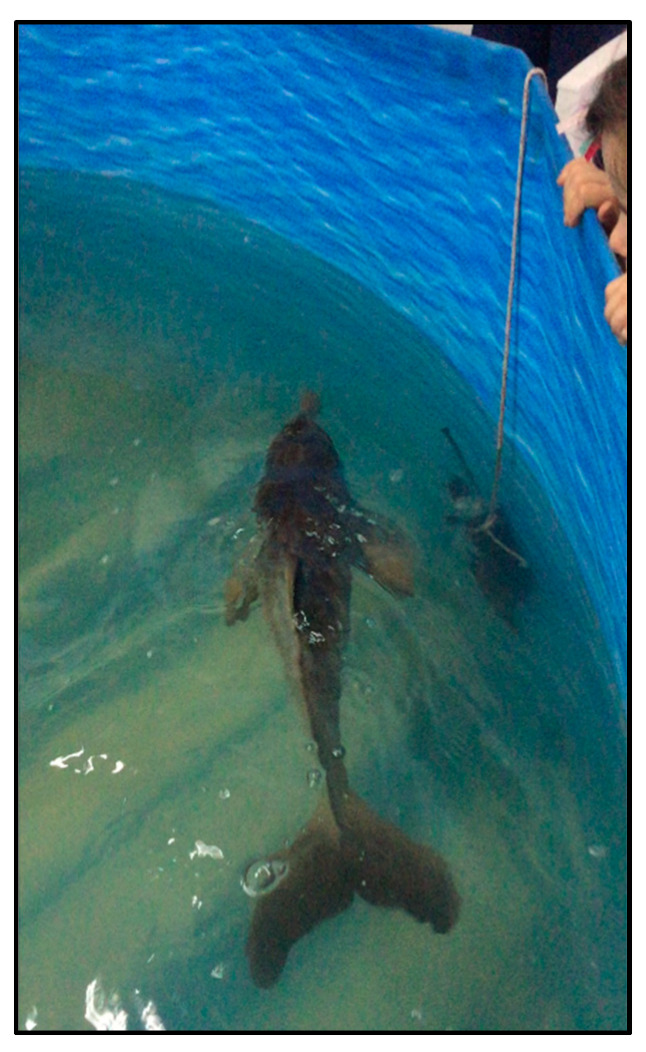
Neonate of franciscana in the rehabilitation centre of Fundación Mundo Marino in San Clemente del Tuyú, Buenos Aires, Argentina.

The first neonate was a 68 cm long male, weighing 4.1 kg, that entered the rehabilitation centre on 13 November 2021 and died two days after being brought into the facility. It was kept in a circular holding pool (3.5 m diameter and 1 m deep) in sea water maintained at 25 °C, with a salinity of 20‰. The animal had wounds near the anus and around the left eye. It was diagnosed with acute suppurative bronchopneumonia and showed an irregular swimming pattern, tilted with the right side of the body towards the surface (Figure 2). Based on the growth curve for franciscanas [35], it was estimated that this individual was less than one month old.

Acoustic recordings began on 14 November at 19:30 (local time), recording continuously for 22 h.

#### 2.1.2. Neonate 2

The second neonate, also a male, was transported to the rehabilitation centre on 9 December 2021 and placed in the same holding pool. It was also 68 cm in length and 4.3 kg in weight. It was diagnosed with anaemia and hepatitis, surviving only three days. Acoustic recordings began on the 10th at 13:30 (local time), recording continuously for 28 h. Based on the growth curve for franciscanas [35,36], it was estimated that this individual was under one month old.

#### 2.1.3. Juvenile

On 29 July 2022, a juvenile female franciscana (estimated to be about one to two years old, [35,36]) was admitted to the rehabilitation centre and placed in a circular tank, 13 m in diameter and 1.35 m deep. The water level was kept constant, with an average temperature of 26.2 °C and a salinity of 23‰. She measured 93 cm long and weighed 10.4 kg. Acoustic recordings were taken during three periods from 29 July to 3 August, from 31 August to 5 September, and from 5 to 12 September, for 252 h. The dolphin died 68 days later, on 6 October.

#### 2.1.4. Wild Animals

On two separate occasions in November 2021, the ST was deployed in two artisanal gillnets in Claromecó (Figure 1), 1.4 nm offshore (depth = 15 m). The fishermen travel in 8 m fiberglass boats and use 50 m long, 1.5 m high monofilament gillnets, with a mesh size of 10.5 cm. Generally, five or six 50 m sections are assembled together; thus, the nets reach 250 to 300 m in length (see [37] for more information). The main target of the fishermen is the Scienids (*Cynoscion guatucupa* and *Micropogonias furnieri*), which are common prey for the franciscana [36,38,39,40].

### 2.2. Data Analysis

The raw audio files were analysed using D-PorCCA version1.2, a standalone software specifically developed to analyse acoustic recordings of NBHF species. The software includes the transient sound detector from PAMGuard version 1.15.10 [41], the PorCC version 1.2 classifier [42], and a click train detector that works by grouping clicks based on repetition rates and amplitude variations. The outputs of D-PorCCA include a list of all click trains detected in the data, with information such as date, time, number of clicks, and quality of the train, as well as a list of all clicks, including parameters such as duration, centre frequency, and click train (if appropriate).

#### 2.2.1. Click Parameters

A random selection of 100 click trains from the juvenile and all click trains from the neonates and the wild animals were audited, and the best quality samples (see criteria below) were chosen for the subsequent selection of individual clicks to estimate the following parameters:

- Peak frequency (kHz): frequency with highest amplitude in the power spectrum of the click.
- Centre frequency (kHz): estimated as the point dividing the spectrum in halves of equal energy, derived by the squared pressure over a 256-point (128 µs) window, symmetrical surrounding the peak of the signal envelope [43].
- Bandwidth (−3 dB): defined as the bandwidth around the peak frequency that contains half of the signal power [44].
- Bandwidth (rms): the root mean square bandwidth is defined as the spectral standard deviation around the centroid frequency on a linear scale (algorithm courtesy of J. Tougaard [44]).
- Duration (µs): estimated as the 80% energy of the clip that contains the signal [44].

The parameters were estimated from clips of the same duration (301 samples, 522 µs), with the click at the centre of the clip. Subsequently, all clicks in the selected click trains were audited using the criteria below, employing custom-built standalone desktop applications, built using the AppDesigner environment in MATLAB 2018a (Mathworks, Natick, MA, United States) to manually classify the selected clicks, as well as to delete echoes and other sources of sounds. The application simultaneously displays the waveform, power spectrum, and spectrogram of the click being audited, as well as the amplitude and centroid frequency [45].

#### 2.2.2. Auditing—Click Trains

The click trains were audited by inspecting the normalized amplitude of the clicks, the spectrogram, and the repetition rate variation throughout the train. At least three of the four criteria below had to be met for the train to be selected for further analysis:

1. At least 10 clicks.
2. Clicks with good signal-to-noise ratio and clearly visible in the spectrogram.
3. Low background noise levels, specially at frequencies over 50 kHz.
4. Non-distorted waveform of individual clicks.

#### 2.2.3. Auditing—Individual NBHF Clicks

The clicks were audited by inspecting the waveform, the power spectrum, and the spectrogram of the original and a filtered click. The original signal was filtered using a bandpass Butterworth filter (order 6), with cutoff frequencies at 25 and 190 kHz. The filters were used as the recordings were made in areas with high levels of low-frequency noise, thus impacting the estimation of the parameters mentioned above.

At least three of the four criteria below had to be met for the click to be selected for further analysis:

1. Good signal-to-noise ratio.
2. No echo or another source of sound present in the clip.
3. No overlap of the click with an echo or another source of sound.
4. Polycyclic, with a bell-like envelope [27].

#### 2.2.4. Classification of Click Train Types

The click trains were analysed using a custom MATLAB application specifically developed for this study. Initially, each WAV file underwent a verification process to ensure data integrity. Subsequently, normalization techniques were applied to standardize the audio amplitude across the recordings, minimizing variations in loudness that could affect analysis results. To focus exclusively on relevant high-frequency components, a high-pass filter, with a cutoff frequency of 50,000 Hz, was implemented. This filter effectively attenuated lower-frequency noise, ensuring that only high-frequency click signals were analysed. The MATLAB application systematically scanned each processed WAV file to detect the start samples of all clicks. This detection process was carefully calibrated to pinpoint the exact onset of each click within predefined sample ranges. The results of this detection were logged in an Excel spreadsheet, which included columns for click number, WAV file identifier, and the corresponding start sample number. Once the start samples of clicks were identified and recorded, the next step involved calculating the inter-click intervals (ICI). This metric provides insights into the temporal spacing between consecutive clicks. Additionally, the MATLAB application computed the clicks per second (CPS) as a measure of the click rate within the audio recordings. Throughout the analysis process, the calculations for ICI and CPS were uniformly applied across all detected clicks within the dataset. This approach ensured consistency and accuracy in the derived metrics, facilitating robust comparative analyses and interpretations. The MATLAB application facilitated the visualization of click trains through waveform displays and spectrograms, as follows:

Waveform: the waveform visualization illustrated the amplitude variation of the click signals over time, providing a direct representation of their temporal distribution and intensity.

Spectrogram (FFT: 512): the spectrogram displayed the frequency content of the audio signal over time, using a fast Fourier transform (FFT) with a window size of 512 samples.

#### 2.2.5. Low-Frequency Clicks and Whistles

All raw audio files were audited in Raven Pro 1.4 (Cornell Lab of Ornithology, Ithaca, NY, USA) with a Hann window of 512 points, with the purpose of identifying whistles and clicks with an energy content in frequencies below 100 kHz by simultaneously looking at the waveform and the spectrogram.

### 2.3. Statistical Analysis

All statistical analyses were performed in the programming environment R [46]. In order to compare the click parameters between categories (neonate, juvenile, unknown), a nonparametric test for non-normally distributed data was performed (Kruskal–Wallis ANOVA), and a post hoc (Tukey’s range test:) analysis was employed using the *pgirmess* package in R. Additionally, a linear discriminant analysis (LDA) employing the *Mass* package and visualization using the *ggord* package were performed to investigate the potential of using acoustic features to discriminate between age categories, namely neonates, juveniles, and those of unknown age. The analysis of similarities (ANOSIM), using the Bray–Curtis dissimilarity measure, was employed to uncover the distinctive acoustic communication patterns among neonates (Calf), the juvenile (Juvenile), and wild dolphins with no class id subgroup (Unknown)

## 3. Results

A total of 1170 min were analysed for neonate 1 (77 click trains), 1678 min for neonate 2 (62 click trains), 15,120 min for the juvenile (100 click trains), and 1410 min for the animals in the wild. No whistles or clicks with energy concentrated below 100 kHz were found in the data for any of the animals.

### 3.1. NBHF Click Parameters

**Figure 3:**
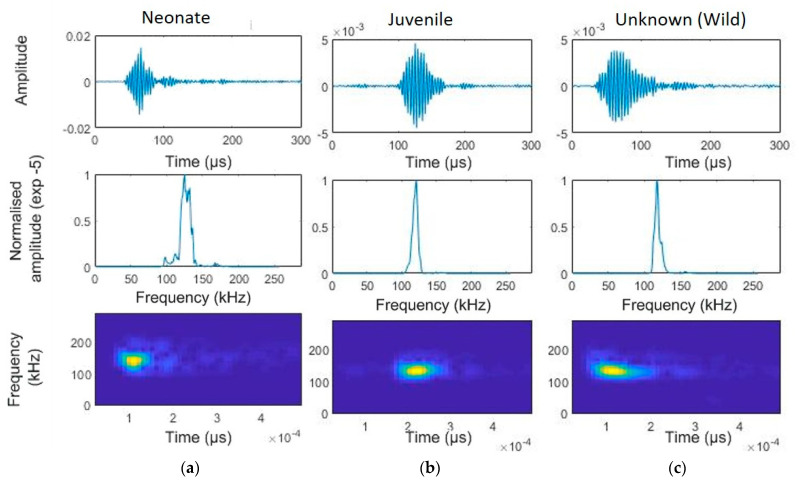
Temporal characteristics of a narrow-band high-frequency click of a selected franciscana dolphin using the app developed by Dr. Mel Cosentino, created using the MATLAB (Mathworks, Natick, MA, United States) AppDesigner (version 2017a) feature (FFT = 512, window size = 64, overlap 50%). Top panels: waveform (amplitude is normalised using the clipping level of the hydrophone as the maximum value). Central panels: normalized power spectral density. Bottom panels: smoothed pseudo Wigner–Ville distribution (FFT = 512, window size = 64, overlap 50%): (**a**) neonate, (**b**) juvenile, and (**c**) unknow age, from wild dolphins near the gillnets.

A total of 12,176 individual clicks were audited for all animals, and these were part of selected click trains (Figure 3). This resulted in 513 clicks, from which several parameters were estimated (Table 1). Differences among age categories and between captive and wild individuals were observed in regards to bandwidth, frequency characteristics, and click duration. Neonates exhibited broader bandwidths (−3 dB) compared to those of the juvenile and wild individuals. Peak and centre frequencies also showed variability across categories, with the juveniles generally demonstrating narrower bandwidths and more consistent centre frequencies. Detailed statistical values, including means, standard deviations (SD), interquartile ranges (IQR), coefficients of variation (CV), and minimum and maximum values for each parameter, are summarized in Table 1.

**Figure 4:**
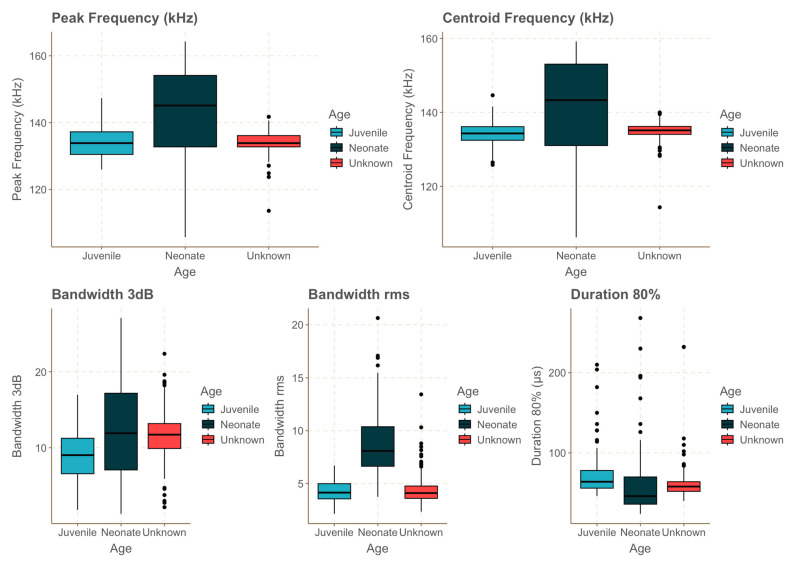
Boxplots showing variations in several click parameters for neonates (dark blue), the juvenile (light blue), and wild individuals of unknown age (red). Black circles represent outliers in the data.

All the between-group comparisons were statistically significant, except for BWrms between the juvenile and wild animals, BW−3 dB between the wild animals and neonates, and peak frequency between the juvenile and wild animals. Neonates showed a significantly wider −3 dB bandwidth compared to those of the juvenile and wild individuals. Neonates also exhibited a significantly higher peak and centre frequencies compared to those of both the juvenile and wild individuals, as well as a significantly higher rms bandwidth. The juvenile exhibited the longest click lengths compared to those of the neonates and wild individuals (Figure 4, Table 2). In addition, the neonates presented greater dispersion of the data in regards to the peak frequency, centre frequency, and bandwidth (Figure 4).

**Figure 5:**
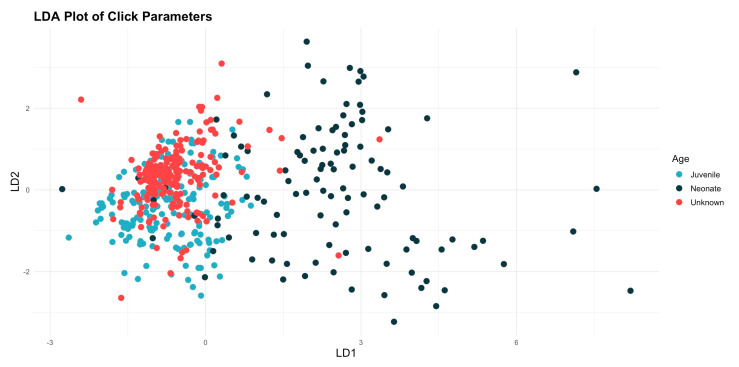
Plot illustrating the results of the linear discriminant analysis (LDA), showing distinct a separation between the results for the neonates and those from the other two categories, while the results for the unknowns were more closely association with the juvenile.

The linear discriminant analysis (LDA) showed that there were differences between the categories, and the results for the wild animals were closer to those of the juveniles than those of the neonates (Figure 5). The subsequent analysis of similarities (ANOSIM), assessed using Bray–Curtis similarity, indicates that there are significant differences between the age categories. The value of the ANOSIM statistic is 0.341, and the significance is 0.001, suggesting that the observed differences are not the result of chance.

**Figure 6:**
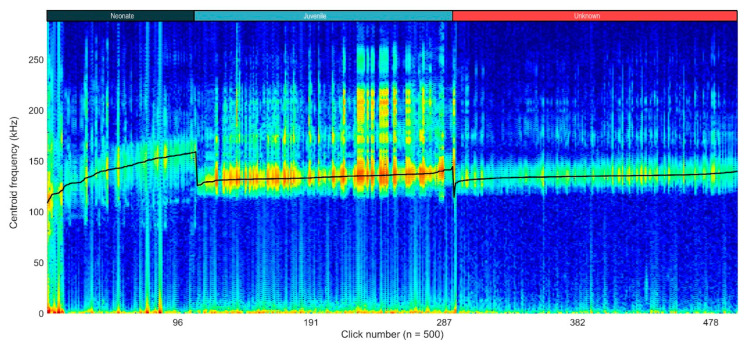
Concatenated spectrogram of all selected clicks (n = 500). The clicks are sorted by centre frequency, in ascending order, per age group. The black line represents the centroid frequency (in kHz). Drops in the black line represent changes in the age group: neonates (n = 2), juvenile (n = 1), unknown (obtained from an unknown number of wild animals).

Moreover, the variation in click characteristics across different age categories can be visualized in the concatenated spectrogram (Figure 6). The distinct separation in centroid frequencies highlights the acoustic differences between neonates, juveniles, and wild animals of unknown age. The gradual increase in the centre frequency within each age group and the noticeable drops at the transitions suggest age-related acoustic variations, with neonates exhibiting higher centroid frequencies compared to the juveniles and unknown wild individuals.

### 3.2. Classification of Click Train Types

**Figure 7:**
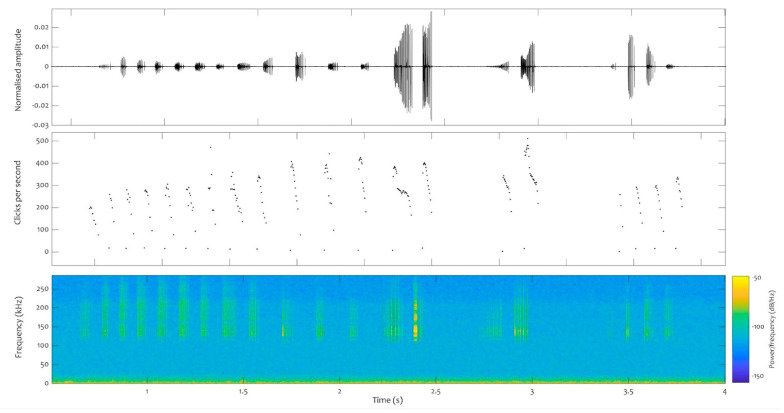
Click train (burst pulses) recorded from the juvenile on the 2 September 2022, just after 2 pm (local time). Top panel: waveform. Middle panel: repetition rates in clicks per second. Bottom panel: spectrogram (FFT: 512). Y axis: time in seconds.

A total of 77 click trains were analysed for neonate 1, 62 for neonate 2, and 629 for the juveniles. At least two consistent repetition patterns were found in all the files analysed, both in those obtained from species in captivity (neonate and juvenile) and from dolphins in the wild. One of these patterns exhibits a downward sweep, with fewer than 200 repetitions per second, while the other pattern involves a series of high repetition rate clicks that sharply drops from 500 repetitions per second onwards, with decreasing repetition rates emitted in bouts (Figure 7).

## 4. Discussion

This study is the first to collect such an extensive amount of recording hours using a high-frequency hydrophone for franciscana dolphins, not only in Argentina but throughout their entire distribution range. The results of this study confirm that franciscanas emit clicks of a narrow band and high frequency nature, with characteristics similar, but not identical, to those previously reported for this and other species [16,18,25,26,27,47] (Table S1). The use of the same device to record franciscanas of the same and different age categories, both in human care and in the wild, made it possible to directly compare the acoustic characteristics between categories. One of the most important discoveries in this study is that the acoustic characteristics of neonate clicks are significantly different from those of the juvenile and wild unknow age franciscana dolphins, providing an opportunity to develop acoustic classifiers specifically for this age group. The ability to detect young individuals is required for the protection of this species because it would allow for the identification of critical habitats or seasons for franciscanas, due their pulsatile pattern of births [35,48]. In Anegada Bay, Argentina franciscana dolphins move inshore during spring and summer for feeding, mating, and calving [4]. This behaviour suggests that coastal areas may offer refuge to calves and females during the reproductive period, providing protection from predation in a food-rich habitat. Franciscanas are preyed upon by both sharks and killer whales [49,50,51]; therefore, is possible that they use coastal and shallow habitats in the calving season as a strategy to avoid encounters with predatory sharks, a behaviour that has been proposed for other cetaceans [52,53].

The sounds of the juvenile, as well as dolphins of unknown age, recorded around gillnets in the wild exhibited similar acoustic characteristics. While the age of the dolphins around the gillnets recorded in this study is unknown, in Argentina, most bycaught animals ensnared in these types of nets are juveniles [8,54,55], which explain these similarities. On the other hand, it is also possible that the clicks emitted by juveniles are similar to those emitted by adults, which are also found around gillnets [8].

The first acoustic recording of a neonate franciscana, of the same length as the ones studied here, was produced over three hours in a tidal pool in the Rio Negro estuary (Argentina). The researchers determined a minimum peak frequency of 37.3 kHz and a maximum of 160.3 kHz [26]. However, it is difficult to assess the nature of those low frequencies, given the information provided by the authors, as there is no spectrogram of low-frequency click trains, and the concatenated spectrogram is not sufficient to show that there are two types of clicks. The other records of neonate franciscanas are from Uruguay, which were also recorded in captivity [28], but as the equipment used to record these sounds did not cover frequencies over 100 kHz (without any mention of use of an aliasing filter), the results are not directly comparable. Despite using different equipment, these authors also suggest that neonatal dolphins generally exhibit a wider bandwidth, which is likely due to their developmental stage rather than resulting from an abnormal state or disease, as suggested by Frainer et al. [56]. Even though our dolphins of known age were undergoing rehabilitation, we manually selected a substantial number of clicks that remained consistent in their characteristics and that did not show alterations in their form, according to the specific criteria described in the Methods section, and applied these criteria to healthy *Phocoena phocoena* [45]. Generally, as dolphins approach death, they tend to decrease their click production rate rather than modify their acoustic parameters, as we noticed in common dolphins [57].

Here, for the first time, we reported different click repetition patterns in franciscana dolphins, which could indicate communication. In other non-whistling species that produce NBHF clicks, such as the harbour porpoise (*Phocoena phocoena),* the rate at which clicks are produced appears to be linked to the behaviour of the animal. While inspecting the environment, this species generally emits between 20 to 60 clicks per second, but this rate increases to almost 100 clicks per second when the porpoise is approaching an object [58,59,60] and to several hundred clicks per second when it is feeding, in what is known as a feeding buzz [61]. Therefore, we anticipated that the production rate of clicks would vary, depending on the specific behaviour exhibited by the emitting dolphin. Given the fact that the franciscana in rehabilitation did not have access to free-swimming fish, it is possible that the high-repetition sounds are some form of social noise, as previously suggested by Melcon et al. [26] for calves in the tide pool.

Traditionally, feeding buzzes have been identified by estimating the mean repetition rate of the train. If this rate exceeds 100 clicks per second, then it is considered a feeding buzz. However, this is not a reliable methodological approach, as burst pulses also have exhibit repetition rates, and these do not indicate feeding, but communication. On the other hand, using 100 clicks per second as a threshold to identify burst pulses, as did Barcellos et al. [30], is also inadequate. Data from other NBHF species, especially the harbour porpoise, show that while there are differences in repetition rates between social sounds, foraging buzzes, and normal echolocation [62,63], the differences are not clear-cut, and more data on the context of these sounds is needed.

Here, we found at least two distinct patterns of click repetition for franciscana dolphins, one of which could it be similar to those called “packet sounds” by Terada et al. [24], as noted in captive narrow-ridged finless porpoises (*Neophocaena asiaeorientalis*), based on spectrogram observations. However, the authors did not provide illustrations of clicks per second to directly compare whether the forms are similar. Moreover, our high repetition click patter follows an opposite direction compared with those found in the harbour porpoise *Phocoena phocoena,* designated as contact calls [62]. Porpoises produce these sounds up to 27 times per minute and primarily during social interactions [63]. Like porpoises, franciscanas may use these click trains for communication during social interactions, although further research is needed to confirm this hypothesis and to establish clear associations between the repetition click patterns in franciscana and specific daily activities, such as feeding or interactions with caregivers. Understanding the role of these sounds in franciscanas could provide deeper insights into their social structures and communication strategies

We did not find any whistles or clicks exhibited an energy level entirely below 100 kHz. Previous research in Brazil documented the occurrence of whistles in franciscanas, mainly during capture, tagging, and release procedures, which are unusual and occur during stressful situations [29]. The study suggested that the sounds might have been produced by the calf, or possibly by the mother to maintain contact with the calf [64]. However, we did not record any whistles in the two neonates studied. The role of whistles as contact calls, particularly between mother and calf, has also been suggested for other NBHF dolphins, such as Commerson’s dolphins (*C. commersonii*) [20]. It is plausible that the whistles documented by Cremer could have been produced by females as contact or warning signals for their calves, especially given that both calves were in the suckling phase during the capture/tagging/release procedures [29]. The absence of such signals in our recordings could be due to the different contexts in which the animals were studied.

Finally, aquariums and zoos play a significant role in advancing our understanding of marine mammals, particularly for species that are challenging to study in their natural habitats. These facilities allow for the recording of species that cannot be easily recorded in the wild, such as franciscanas and the spectacled porpoise *Phocoena dioptrica* [65], among others. Through these contributions, aquariums and zoos are essential for enhancing our knowledge and conservation efforts for marine mammal species like the franciscana dolphin.

## 5. Conclusions

This study examined the acoustic characteristics of franciscana dolphins (*Pontoporia blainvillei*), comparing individuals in captivity with those in the wild. The consistent use of the same recording device for both captive and wild dolphins enabled direct comparisons, revealing that neonate clicks are markedly different from those of the juvenile and wild individuals near the gillnets. Neonates exhibited sounds with broader bandwidths and higher peak frequencies compared to those of the juvenile and wild individuals. Further analysis identified consistent repetition patterns across all age categories, including a downward sweep pattern and high-repetition-rate clicks. These findings are important for developing new age-specific acoustic classifiers which can help to identify critical habitats or seasons essential for the protection of franciscanas.

Furthermore, the juvenile and wild dolphins recorded near gillnets exhibited similar acoustic traits, likely due to the high incidence of juvenile bycatch in these nets. These results enhance our understanding of franciscana dolphin communication and emphasize the value of acoustic monitoring for conservation and management efforts.

## Figures and Tables

**Table 1:** Click parameters of *Pontoporia blainvillei* from two neonates (n = 109) and one juvenile (n = 198) held in rehabilitation, and from animals recorded around gillnets in the wild (n = 206). SD = standard deviation; IQR = interquartile range; CV = coefficient of variation; Min = minimum value; Max = maximum value.

	Mean	SD	IQR	CV	Min	Max
Bandwidth −3 dB (kHz)						
Neonates	12.7	6.5	10.1	0.5	1.3	27.0
Juvenile	9.0	3.2	4.7	0.4	1.8	17.0
Unknown	11.6	3.0	3.3	0.3	2.2	22.4
Bandwidth rms (kHz)						
Neonates	8.8	3.2	3.7	0.4	3.7	20.6
Juvenile	4.2	1.0	1.4	0.2	2.1	6.7
Unknown	4.4	1.3	1.2	0.3	2.3	13.4
Centre frequency (kHz)						
Neonates	141.4	13.3	22.0	0.1	106.2	159.2
Juvenile	134.4	3.0	3.7	0.0	125.8	144.6
Unknown	135.0	2.5	2.1	0.0	114.3	140.0
Peak frequency (kHz)						
Neonates	141.8	14.7	21.4	0.1	105.8	164.3
Juvenile	133.9	4.2	6.8	0.0	126.0	147.4
Unknown	134.4	3.5	3.4	0.0	113.6	141.8
Duration 80% (µs)						
Neonates	61.2	43.5	34.0	0.7	24.0	268.0
Juvenile	70.0	23.4	22.0	0.3	46.0	210.0
Unknown	60.4	16.6	12.0	0.3	40.0	232.0

**Table 2:** Multiple comparison test (Tukey’s range test) using the Kruskal–Wallis results. Observed diff: differences between the medians of each group; Critical diff = the difference between age categories to qualify as statistically significant; Significance = if Observed diff > Critical diff, then it is statistically significant and marked with an asterisk (*) with a 95% confidence level (*p* < 0.05); N.S. = not significant.

Compared Age Categories	Observed Diff	Critical Diff	Significance
−3 dB Bandwidth (kHz)			
Neonates—Juvenile	99.9	42.3	*
Neonates—Unknown	7.3	42.0	N.S.
Juvenile—Unknown	107.2	35.3	*
RMS Bandwidth (kHz)			
Neonates—Juvenile	233.8	42.3	*
Neonates—Unknown	229.8	42.0	*
Juvenile—Unknown	3.9	35.3	N.S.
Centre frequency (kHz)			
Neonates—Juvenile	123.1	42.3	*
Neonates—Unknown	87.1	42.0	*
Juvenile—Unknown	36.6	35.3	*
Peak frequency (kHz)			
Neonates—Juvenile	115.1	42.3	*
Neonates—Unknown	93.6	42.0	*
Juvenile—Unknown	21.5	35.3	N.S.
Duration 80% (µs)			
Neonates—Juvenile	120.8	42.3	*
Neonates—Unknown	51.0	42.0	*
Juvenile—Unknown	69.8	35.3	*

## Data Availability

The datasets generated for this study will not be made publicly available. However, the data may be available upon request with appropriate permissions.

## Supplementary Materials

The following supporting information can be downloaded at: https://www.mdpi.com/article/10.3390/ani14162436/s1. References [18,47,66] are cited in the Supplementary Materials.
