# Supplementary material for: Detailed Comparison of Acoustic Signals from Rehabilitated and Wild Franciscanas (Pontoporia blainvillei) Dolphins
Source: Animals (Basel). 2024 Aug 22;14(16):2436. doi: 10.3390/ani14162436 (PMC11350683; doi:10.3390/ani14162436)
Supplement: Supplementary file 1 [file animals-14-02436-s001.zip › animals-3131295-supplementary.pdf]

Detailed acoustic signals of franciscanas (*Pontoporia blainvillei*) comparison of rehabilitated and wild dolphins

Giardino, Gisela [1] *, Mel Cosentino [2], Agustina Macchi [1], Juan Pablo Loureiro [3], Sergio Rodriguez Heredia [3],Karina Alvarez [3], Sergio Moron [3] and Diego Rodriguez ,[1]

Table S1.

**Parameter**	**Peak frequency (kHz)**	**Centroid frequency (kHz)**	**3 dB bandwidth (kHz)**	**rms bandwidth (kHz)**	**Source**
**South American NHBF cetacean**	Mean ± stdev (range)	Mean ± stdev (range)	Mean ± stdev (range)	Mean ± stdev (range)	
Neonate Franciscana dolphins (*Pontoporia blainvillei*)	141.8± 14.7 (105.8-164.3)	141.4± 13.3 (106.2-159.2)	12.7± 6.5 (1.3-27)	8.8± 3.2 (3.7-20.6)	Present work
Juvenile franciscana dolpnin (*Pontoporia blainvillei*)	133.0± 3.0 (126.0-147.4)	134.0± 3.0 (125.8-144.6)	9.0±3.2 (1.8-17.0)	4.2± 1.0 (2.1-6.7)	Present work
Burmeister's porpoise (*Phocoena spinipinnis*)	138 ± 11 (129–186)	146 ± 8 (134–178)	8 ± 2 (6–11)		[1]
**Other NBHF centaceans**					
Hector's dolphin (*Cephalorhynchus hectori*)	129 ± 5 (117-135)	128 ± 3 (125-132)	20 ± 3 (12-26)	18 ± 5 (11-29)	[2]
Hourglass dolphin (*Lagenorhynchus cruciger*)	126 ± 2 (122-131)	128 ± 2 (124-132)	8 ± 2 (5-11)	11 ± 4 (5-22)	[2]
BC Dall's porpoise (*Phocoenoides dalli)*	137±64 (119–143)	137±63 (121–147)	11±5 (3-23)	8±2 (5-14)	[3]

BC Harbour porpoise (*Phocoena phocoena*)	140±61 (137–143)	141±62 (138–148)	8±3 (3–19)	8±2 (5–14)	[3]
Danish harbour porpoise (*Phocoena phocoena*)	137±66 (112–145)	136±63 (126–144)	17±65 (5–36)	10±62	[3]

1. Reyes, V.; Marino, A.; Dellabianca, N.A.; Hevia, M. Clicks of Wild Burmeister's Porpoises (Phocoena Spinipinnis) in Tierra Del Fuego, Argentina: NOTES. *Marine Mammal Science* **2018**, doi:10.1111/mms.12489.
2. Kyhn, L.A.; Tougaard, J.; Jensen, F.; Wahlberg, M.; Stone, G.; Yoshinaga, A.; Beedholm, K.; Madsen, P.T. Feeding at a High Pitch: Source Parameters of Narrow Band, High-Frequency Clicks from Echolocating off-Shore Hourglass Dolphins and Coastal Hector's Dolphins. *The Journal of the Acoustical Society of America* **2009**, *125*, 1783–1791, doi:10.1121/1.3075600.
3. Kyhn, L.A.; Tougaard, J.; Beedholm, K.; Jensen, F.H.; Ashe, E.; Williams, R.; Madsen, P.T. Clicking in a Killer Whale Habitat: Narrow-Band, High-Frequency Biosonar Clicks of Harbour Porpoise (Phocoena Phocoena) and Dall's Porpoise (Phocoenoides Dalli). *PLoS ONE* **2013**, *8*, e63763, doi:10.1371/journal.pone.0063763.
